# FeNO as a Non-Invasive Biomarker of Type 2 Inflammation in Chronic Rhinosinusitis with Nasal Polyps: Correlation with Serum IgE and Eosinophils

**DOI:** 10.22038/ijorl.2025.88968.3979

**Published:** 2026

**Authors:** Sahar Fereydouni, Farahzad Jabbari-Azad, Mehdi Bakhshaee, Maryam Khoshkhui, Yaser Yadegari, Mojgan Mohammadi

**Affiliations:** 1 *Allergy Research Center, Mashhad University of Medical Sciences, Mashhad, Iran.*; 2 *Sinus and Surgical Endoscopic Research Center, Mashhad University of Medical Sciences, Mashhad, Iran. *

**Keywords:** Blood eosinophil, CRSwNP, Chronic rhinosinositis, FeNO, IgE, Nasal smear

## Abstract

**Introduction:**

Chronic rhinosinusitis with nasal polyps is a heterogeneous inflammatory disorder often driven by type 2 inflammation. Identification of non-invasive biomarkers, such as fractional exhaled nitric oxide (FeNO), may support the assessment and personalized management.

**Materials and Methods:**

In this cross-sectional study, 141 patients with clinically and radiologically confirmed CRSwNP were assessed. FeNO levels, nasal eosinophilia, serum IgE, peripheral blood eosinophil counts, and skin prick test results were evaluated. Disease severity was measured using the SNOT-22 and Lund-Mackay CT scores.

**Results:**

FeNO showed a significant correlation with nasal eosinophilia (r = 0.53, p < 0.001), a moderate correlation with serum IgE (r = 0.40, p < 0.001), and a weak correlation with blood eosinophils (r = 0.17, p = 0.047). Serum IgE was significantly associated with both SNOT-22 and Lund-Mackay scores. FeNO did not show significant correlation with symptom severity or radiological extent, suggesting its use as a supportive marker rather than a stand-alone predictor.

**Conclusion:**

FeNO may serve as a potential non-invasive marker for type 2 inflammation in CRSwNP, though it may not predict disease severity. Combining FeNO with other markers could improve clinical endotyping and management.

## Introduction

Chronic rhinosinusitis with nasal polyps (CRSwNP) is a persistent inflammatory condition affecting the mucosal lining of the nasal cavity and paranasal sinuses (1). It is considered a common disorder, Overall, approximately 20% of patients with chronic rhinosinusitis have CRSwNP (2). The condition presents with typical symptoms such as nasal obstruction, mucopurulent discharge, facial pressure, and olfactory dysfunction, persisting for at least 12 consecutive weeks according to the European Position Paper on CRS and nasal polyps (EPOS 2020) (3). The chronic nature of the disease and its long-lasting symptoms significantly impair patients' quality of life (4). 

CRSwNP is a heterogeneous disorder with different endotypes, primarily classified as type 2 and non-type 2 inflammation. This classification has clinical relevance, as it may relate to variations in symptom severity, response to treatment, and risk of recurrence (5,6). Type 2 inflammation is typically associated with eosinophilic infiltration, elevated serum IgE levels, and comorbid allergic conditions such as asthma (7,8). The prevalence of type 2 and non-type 2 inflammation in CRSwNP differs among populations, with Asian patients exhibiting more neutrophilic inflammation, whereas type 2 inflammation predominates in Western populations (9).

According to the EPOS 2020 guidelines, type 2 CRSwNP can be defined using specific thresholds: ≥10 eosinophils per high-power field in nasal tissue or peripheral blood eosinophils ≥250 cells/µL, or serum IgE levels ≥100 IU/mL (3). Patients with this endotype may exhibit refractory disease with a high risk of recurrence, despite medical or surgical interventions (2,10,11). The clinical course is variable and unpredictable, making individualized management challenging.

The identification of reliable, non-invasive biomarkers to assess type 2 inflammation in CRSwNP is essential. Among potential candidates, FeNO has emerged as a convenient marker of eosinophilic airway inflammation and is commonly used in asthma management (10,12). Recent studies indicate that FeNO may also reflect upper airway inflammation in eosinophilic and allergic CRSwNP (13). In these patients, nasal polyps (NPs) are recognized as a stable determinant of increased FeNO levels (15).

Nitric oxide has been extensively studied in CRS, primarily because the upper airways are recognized as the primary source of respiratory NO (14,15), and the paranasal sinuses are identified as the main site of its production (16). 

Although several studies have evaluated the utility of FeNO as a biomarker in diseases such as asthma, its clinical role in patients with CRSwNP remains underexplored. Zhang et al. demonstrated that 29% of patients with CRS accompanied by nasal polyps, who did not have pulmonary disease, exhibited elevated FeNO levels (17). Jeong et al. showed that 30 non-asthmatic, non-atopic patients with CRS with nasal polyps had a significantly higher FeNO than healthy controls (18). Furthermore, recent studies demonstrated that in patients with eosinophilic CRS, FeNO levels correlated with the severity of the CT findings (19,20).

 Given that CRSwNP is a heterogeneous condition with variable disease burden and treatment response, there is a pressing need for reliable, non-invasive markers to assist in diagnosis, endotyping, and disease monitoring. Identification of such markers could reduce dependence on invasive procedures and provide a cost-effective approach for clinical management. If FeNO correlates with traditional markers of type 2 inflammation, such as serum IgE, peripheral blood eosinophilia, and nasal eosinophilia, it may serve as a practical surrogate biomarker.

The aim of this study was to evaluate the correlation between FeNO levels and markers of type 2 inflammation, including serum IgE, peripheral blood eosinophil count, and nasal eosinophilia in patients with CRSwNP. Additionally, we sought to determine whether FeNO could serve as a non-invasive surrogate marker for assessing type 2 inflammation in these patients.

## Materials and Methods

### Study Design and Participants

This observational cross-sectional study included 141 patients diagnosed with CRSwNP based on clinical and radiological findings according to the EPOS 2020 criteria (10). Patients were recruited from the otolaryngology clinic of Emam Reza Hospital between 2024 and 2025. A control group was not included, as the study primarily aimed to examine correlations between FeNO, serum IgE, and eosinophil counts among patients with CRSwNP rather than to establish diagnostic thresholds.

Inclusion criteria were adults (≥18 years) with a confirmed diagnosis of CRSwNP based on clinical evaluation and CT findings. All participants had discontinued intranasal and inhaled corticosteroids for at least 14 days, oral corticosteroids for at least 4 weeks, and antihistamines for at least 5 days prior to enrollment to minimize drug effects on FeNO and eosinophil measurements.

Exclusion criteria were recent sinus surgery (within the past 6 months), presence of an active sinus or respiratory infection, any chronic systemic disease or malignancy, or inability to comply with study procedures.

### Clinical Evaluation and Symptom Scoring

All participants completed the Sino-Nasal Outcome Test (SNOT-22) to assess symptom severity and quality of life. The SNOT-22 consists of 22 questions, each addressing various aspects of nasal symptoms, facial pain/pressure, and quality of life. Each question is rated on a scale from 0 to 5. The total score ranges from 0 to 110, with higher scores indicating greater symptom severity and worse quality of life. In this study, SNOT-22 scores ≥40 were considered indicative of severe symptoms (21).

Computed tomography (CT) of the paranasal sinuses was performed for each patient, and disease severity was scored using the Lund-Mackay scoring (LMS) system. This system assigns scores based on the extent of sinus involvement, with a total score ranging from 0 to 24. In this study, LMS ≥12 was considered indicative of severe disease (22). 

### Laboratory Tests and Biomarker Assessment

#### FeNO measurement:

FeNO levels were measured using the NObreath® device (Bedfont® NObreath®), a portable, battery-operated analyzer suitable for use in both adults and children. Patients performed a single-breath exhalation at a controlled flow rate of 50 mL/s, following standardized ATS/ERS recommendations, with visual feedback provided by the device to ensure reproducibility. Each patient performed repeated exhalations until two values agreed within 10%, and the mean of these values was recorded as the FeNO. Levels were expressed in parts per billion (ppb). According to ATS guidelines, FeNO levels <25 ppb are considered normal, 25–50 ppb intermediate, and >50 ppb high (23). Intermediate and high FeNO values are indicative of eosinophilic and type 2 airway inflammation, which are frequently observed in CRSwNP. Patients were instructed to refrain from eating, drinking, smoking, or exercising at least 2 hours prior to testing. Use of inhaled or oral corticosteroids was suspended before the measurement according to clinical recommendations (24). 

### Serum Total IgE Levels:

Serum IgE levels were measured via enzyme-linked immunosorbent assay (ELISA). According to EPOS 2020, serum IgE levels ≥100 IU/mL are consistent with eosinophilic CRSwNP (Type 2) (10).

### Peripheral Blood Eosinophil Count:

Peripheral blood eosinophils count were obtained from routine complete blood counts. The normal range for eosinophils is typically <450 cells/μL. In this study, eosinophil counts ≥250 cells/μL were considered elevated, which aligns with the EPOS 2020 threshold for eosinophilic inflammation. Elevated eosinophil counts are commonly associated with type 2 CRSwNP inflammation.

### Nasal Eosinophil Smear:

Nasal cytology was performed for each patient using a cotton swab with gentle rotating movements in the middle portion of the inferior turbinate. Smears were stained with Hansel’s stain and examined under a microscope. Although tissue biopsy is the gold standard for assessing eosinophilic infiltration, nasal smears provide a non-invasive alternative for routine clinical evaluation. A nasal eosinophil count ≥10 cells/HPF was considered significant, according to EPOS 2020 criteria for eosinophilic CRSwNP (10). The limitations of this method, including the potential underestimation of deeper tissue eosinophilia, were acknowledged.

### Skin Prick Test:

Skin prick testing was performed using a standard panel of common aeroallergens. in adults, the test was conducted on the forearm. While in children it was performed on the upper back. After cleaning the test site with alcohol, drops of allergen extract were placed, and each was pricked with a lancet. A wheal ≥3 mm in diameter (compared to saline control) was considered positive, indicating sensitization to that allergen. A positive result reflects sensitization, which may contribute to the allergic inflammation observed in type 2 CRSwNP.

### Ethical Considerations:

The study was approved by the Ethics Committee of Mashhad University of Medical Sciences (approval code: R.MUMS. IRH. REC. 1404.002). Written informed consent and verbal assent were obtained from all patients prior to enrollment and data collection, in accordance with the Declaration of Helsinki.

### Statistical Analysis:

All statistical analyses were conducted using IBM SPSS Statistics version 22.0 (IBM Corp., Armonk, NY, USA). The Kolmogorov–Smirnov test was used to assess the normality of data. Given the non-normal distribution of most variables, non-parametric methods were applied. Spearman’s rank correlation coefficient was used to assess correlations between continuous variables. Group comparisons were conducted using the Mann–Whitney U test for continuous variables and Fisher’s exact test for categorical variables. Chi-square tests were used to examine associations between categorical data. A p-value of <0.05 was considered statistically significant.

## Results

This observational cross-sectional study included 141 patients with CRSwNP diagnosed according to EPOS 2020 criteria. The mean age was 42.12 ± 12.56 years (range 18–77), with a nearly equal gender distribution (50.4% male, 49.6% female).

The mean FeNO level was 26.87 ppb (range 4–70 ppb). Among patients, 56.7% showed normal levels (≤25 ppb), 38.3% had intermediate levels (25–50 ppb), and 5% had high levels (>50 ppb), reflecting different levels of FeNO that may suggest varying degrees of airway inflammation. The mean serum IgE was 233.74 IU/mL (range 6–1300 IU/mL), with 68.1% of patients showing elevated levels (≥100 IU/mL), which may indicate possible allergic sensitization.

The average nasal eosinophil count was 9.69 cells/HPF (median 6, range 2–80). Among patients, 36.2% had nasal eosinophilia (≥10 cells/HPF), reflecting local eosinophilic inflammation. The mean peripheral blood eosinophil count was 587.78 cells/μL (range 20–3450 cell/μL), and 72.3% had elevated counts (≥250 cells/μL), suggesting systemic eosinophilia.

The mean Lund-Mackay score (LMS) was 16.82 (range 7–24), with 77.3% of patients having LMS ≥12, indicating moderate to severe radiological sinus involvement. The mean SNOT-22 score was 39.11 (range 0–72), with 73.8% of patients scoring ≥40, indicating a high symptom burden.

**Fig 1:**
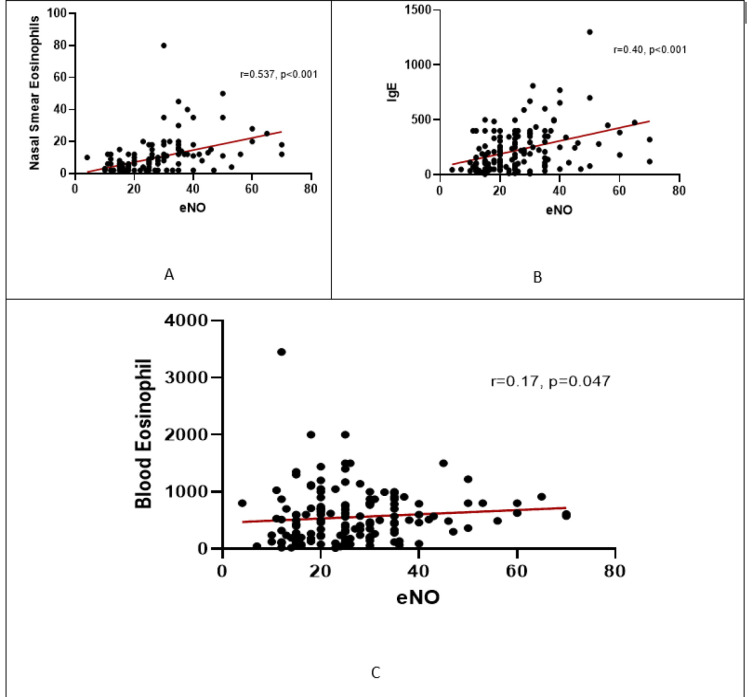
A- Correlation between eNO and Nasal Smear B- Correlation between eNO and IgE levels C- Correlation between eNO and Blood eosinophilia.

A moderate positive correlation was found between FeNO levels and nasal eosinophilia (r = 0.53, p < 0.001; see Figure 1A), suggesting that FeNO may serve as a non-invasive marker of local eosinophilic inflammation. FeNO also showed a moderate correlation with serum IgE levels (r = 0.40, p < 0.001; see Figure 1B), which may reflect an association with allergic inflammation.

A weaker but significant positive correlation was observed between nasal eosinophilia and peripheral blood eosinophils (r = 0.32, p < 0.001). Additionally, FeNO and blood eosinophils showed a mild correlation (r = 0.17, p = 0.047; see Figure 1C), which may indicate a broader inflammatory association.

**Fig 2:**
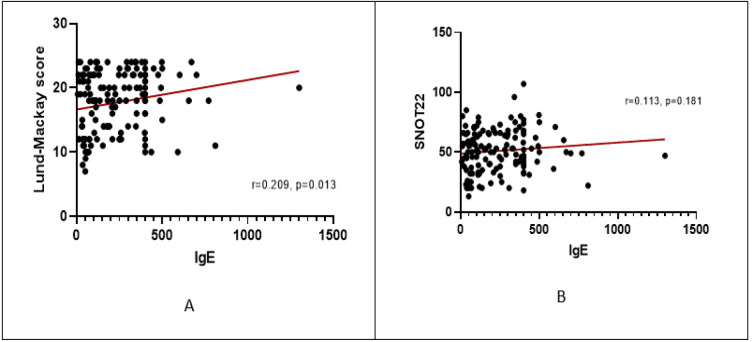
A- Correlation between Lund-Mackay and IgE. B- Correlation between SNOT22 and IgE

Notably, no significant correlation was found between FeNO levels and SNOT-22 symptom severity or LMS (p > 0.05). This suggests that FeNO mainly reflects local eosinophilic inflammation, with limited correlation to overall symptom burden or radiologic disease extent. In contrast, a significant positive correlation was observed between serum IgE levels and the severity of LMS and SNOT-22 (p < 0.001, r = 0.4; see Figures 2A and 2B). 

**Fig 3:**
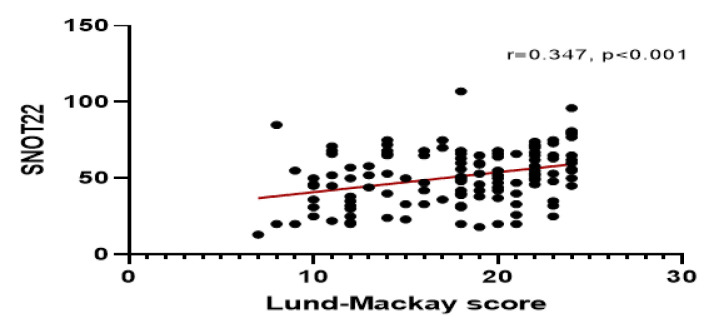
Correlation between SNOT22 and Lund-Mackay score

Additionally, SNOT-22 scores showed a weak but significant correlation with LMS scores (p < 0.001, r = 0.34; see Figure3), indicating that clinical symptoms and imaging reflect partially overlapping but distinct aspects of disease activity. Skin prick testing revealed that 54.6% of patients were sensitized to at least one aeroallergen, with Salsola (saltbush) being the most common (44.7%). 

The most frequent comorbidities were asthma (31.2%) and Samter’s triad (18.2%). Patients with asthma or Samter’s triad had significantly higher SNOT-22 scores (≥40) (p < 0.001), indicating more severe symptoms; however, no significant differences were observed in inflammatory biomarkers between these groups and other patients. 

## Discussion

Our findings suggest that FeNO may serve as a non-invasive, quantitative biomarker for evaluating eosinophilic airway inflammation in patients with CRSwNP. This is consistent with previous reports demonstrating the diagnostic and monitoring value of FeNO in asthma and other type 2 inflammatory airway diseases (25). A comparative study in 2015 on arginine isoforms and CRS phenotypes further supported exhaled nitric oxide as a discriminative marker, reflecting the interplay between arginase and nitric oxide synthase (NOS) activity in NO production (26).

Wang et al. also reported that FeNO levels vary with disease severity, highlighting its potential in early diagnosis and differentiation of sinusitis etiologies (27). Advances in technology have facilitated accurate measurement of nasal NO, enhancing its clinical applicability. In our study, FeNO showed a moderate correlation with nasal eosinophilia, suggesting its potential to identify eosinophilic endotypes in CRSwNP, consistent with finding from Nabavi et al. (28), who reported similar associations. Clinically, FeNO may represent a practical alternative to invasive procedures for patient endotyping.

Moreover, a moderate correlation was observed between FeNO and serum IgE levels, indicating that FeNO may reflect not only local but also systemic type 2 inflammation. This is comparable to findings in asthma research, where Al Ghobain MO et al (29) demonstrated that FeNO can function as a surrogate marker for allergic inflammation. consistent with prior findings (28,30), FeNO levels in our study did not correlate significantly with disease severity as assessed by SNOT-22 or LMS. This suggests that while FeNO effectively captures inflammatory status, it may not reliably reflect symptom burden or radiologic involvement.

Serum IgE levels showed a significant association with both symptom severity (SNOT-22) and radiologic severity (LMS). These findings support previous evidence that serum IgE reflects the extent of systemic allergic and eosinophilic activity contributing to both clinical symptoms and imaging abnormalities (31). 

The significant correlation between SNOT-22 and LMS supports the role of radiologic imaging as a reliable, objective indicator of disease severity that complements patient-reported outcomes (32). In our cohort, 54.6% of patients were sensitized to aeroallergens, with Salsola as the predominant sensitizing allergen. This finding highlight the role of atopy to CRSwNP pathogenesis, particularly in patients with comorbid asthma or Samter’s triad (33). 

These individuals reported higher symptom scores, although their levels of inflammatory biomarkers were not significantly different. Prior research has shown a strong association between CRSwNP and late-onset and treatment-resistant asthma, particularly in older adults (34–37). Although the causal relationship between CRSwNP and asthma remains unclear, the presence of CRSwNP is closely associated with greater asthma severity, especially among older patients (38). Several studies have also demonstrated a significant relationship between asthma severity and the extent of rhinitis or CRS (39).

Furthermore, a significant correlation was observed between peripheral blood eosinophil counts and nasal eosinophilia, suggesting that systemic eosinophilia may reflect localized sinonasal inflammation. This underscores the potential utility of nasal cytology as a simple and minimally invasive, and cost-effective diagnostic tool, especially compared with tissue biopsy (40).

Overall, FeNO appears to be a supportive biomarker for endotyping CRSwNP, particularly for identifying patients with type 2 inflammation who may benefit from targeted therapies. However, given the moderate correlation with nasal eosinophilia and its lack of association with disease severity, FeNO should complement rather than replace other diagnostic markers in clinical evaluation. 

Considering the multifactorial nature of CRSwNP, integration of clinical assessment, imaging finding, and inflammatory biomarkers is essential for accurate diagnosis and personalized management. The cross-sectional design limits causal interpretion, and the absence of a control group may reduce the diagnostic interpretability of FeNO. Moreover, using nasal smear instead of tissue biopsies may have underestimated local eosinophil count. Despite these limitations, our findings support the role of FeNO in CRSwNP endotyping and guiding personalized therapy. However, larger cohort studies and longitudinal research are warranted to assess the independent effects of confounding factors and to confirm the predictive value of FeNO in monitoring treatment response.

## Conclusion

FeNO may serve as a potential non-invasive marker reflecting allergic inflammation and eosinophilia in patients with CRSwNP. However, in our study, FeNO did not correlate with symptom scores (SNOT-22) or radiological findings (LMS), suggesting limited utility in assessing overall disease severity. Given its non-invasive nature and potential role in identifying type 2 inflammation and disease endotypes, further controlled and longitudinal studies are needed to confirm the clinical value of FeNO in the management of CRSwNP. 
